# Reduced cytotoxicity of polyethyleneimine by covalent modification of antioxidant and its application to microalgal transformation

**DOI:** 10.1080/14686996.2021.1978273

**Published:** 2021-10-13

**Authors:** Toru Yoshitomi, Haruka Karita, Natsumi Mori-Moriyama, Naoki Sato, Keitaro Yoshimoto

**Affiliations:** a Department of Life Sciences, Graduate School of Arts and Sciences, The University of Tokyo, Tokyo, Japan; b Research Center for Functional Materials, National Institute for Materials Science, Ibaraki, Japan

**Keywords:** Polyethyleneimine, oxidative stress, reactive oxygen species, transformation, microalgae, antioxidants, 2,2,6,6-tetramethylpiperidine-1-oxyl (TEMPO), polycation, BiomaterialsEnvironment and Sustainability

## Abstract

The conversion of carbon dioxide into valuable chemicals is an effective strategy for combating augmented concentrations of carbon dioxide in the environment. Microalgae photosynthetically produce valuable chemicals that are used as biofuels, sources for industrial materials, medicinal leads, and food additives. Thus, improvements in microalgal technology *via* genetic engineering may prove to be promising for the tailored production of novel metabolites. For the transformation of microalgae, nucleic acids such as plasmid DNA (pDNA) are delivered into the cells using physical and mechanical techniques, such as electroporation, bombardment with DNA-coated microprojectiles, and vortexing with glass beads. However, owing to the electrostatic repulsion between negatively charged cell walls and nucleic acids, the delivery of nucleic acids into the microalgal cells is challenging. To solve this issue, in this study, we investigated microalgal transformation *via* electroporation using polyplexes with linear polyethyleneimine (LPEI) and pDNA. However, the high toxicity of LPEI decreased the transformation efficiency in *Chlamydomonas reinhardtii* cells. We revealed that the toxicity of LPEI was due to oxidative stress resulting from the cellular uptake of LPEI. To suppress the toxicity of LPEI, an antioxidant, 2,2,6,6-tetramethylpiperidine-1-oxyl (TEMPO), was covalently conjugated with LPEI; the conjugate was named as TEMPO-LPEI. Interestingly, with a cellular uptake tendency similar to that of LPEI, TEMPO-LPEI dramatically decreased oxidative stress and cytotoxicity. Electroporation using polyplexes of TEMPO-LPEI and pDNA enhanced the transformation efficiency, compared to those treated with bare pDNA and polyplexes of LPEI/pDNA. This result indicates that polycations conjugated with antioxidants could be useful in facilitating microalgal transformation.

## Graphical abstract

**Figure:**
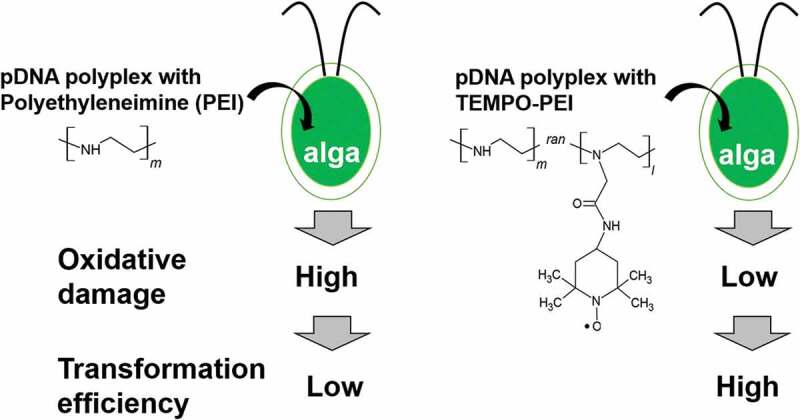

## 1. Introduction

In recent years, increasing concerns about global warming have driven the development of new technologies worldwide to reduce the levels of carbon dioxide in the atmosphere [1]. The conversion of carbon dioxide into valuable products may lower the net cost of removing carbon dioxide from the atmosphere [2]. For example, electrochemical approaches for the conversion of carbon dioxide into alcohols and hydrocarbon fuels have attracted considerable attention; however, these approaches pose various challenges, such as the need for expensive catalysts and low conversion efficiencies [3]. Microalgae have attracted much attention as a promising renewable source that can photosynthetically produce diverse chemicals such as biofuels, sources for industrial materials, recombinant proteins, medicinal leads, antioxidants, and food additives [4–7]. Therefore, improvements in microalgal technology *via* genetic engineering may prove to be promising for the tailored production of novel metabolites [8]. Physical and mechanical techniques such as electroporation, agitation with glass beads, and particle bombardment have been mainly used for microalgal transformation; however, the negative charge of the cell wall [9] makes intracellular delivery of negatively charged macromolecules, such as nucleic acids, difficult due to electrostatic repulsion [10,11]. Therefore, cell-wall-deficient mutant microalgal strains or strains with cell walls removed by enzymatic degradation have often been used for transformation [12]. If there are transforming agents for enhancing intracellular delivery of negatively charged macromolecules, such as nucleic acids, in the native microalgae without removing cell wall, they would be important materials for recombination of microalgae. However, there have so far been no reports on the transforming agents in the field of microalgal researches.

Polycations are used as effective reagents for transformation in a wide range of mammalian cell types because of their capacity to compact nucleic acids into polyion complexes, known as polyplexes, and cellular uptake *via* endocytosis [13–15]. Polyethylenimine (PEI) is a well-known efficient non-viral transfection agent for mammalian cells [16] and has a variety of macromolecular structures (e.g. linear or branched) [17]. The amino groups in PEI are protonated at pH values below their p*K*a, possessing positive charge. Positively charged PEI interacts with negatively charged nucleic acids to form polyplexes [18,19]. A PEI/pDNA polyplex may successfully pass through the negatively charged cell wall and improve the transformation efficiency in microalgal cells.

**Figure 1.**
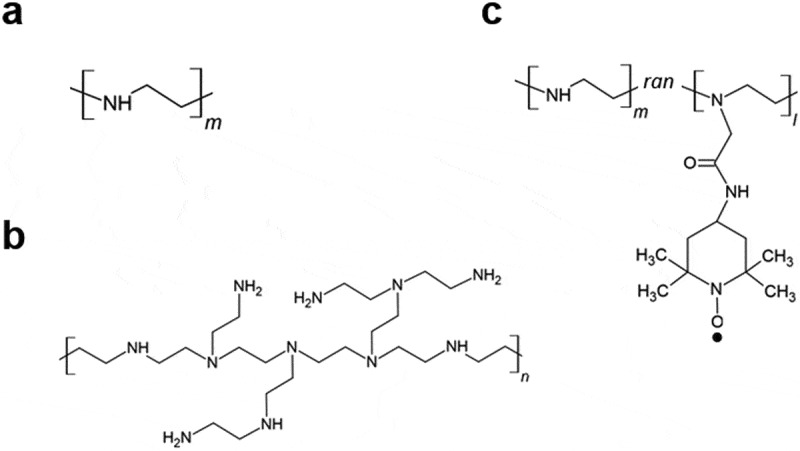
Chemical structure of LPEI, BPEI, and TEMPO-LPEI. (a) Chemical structure of LPEI. The number average molecular weight of LPEI was 10 kDa. (b) Chemical structure of BPEI. The number average molecular weight of BPEI was 10 kDa. (c) Chemical structure of TEMPO-LPEI. The numbers of units in the structure, m and l, were approximately 250 and 2, respectively. The TEMPO moieties were randomly conjugated with the amino groups in the PEI chains

Since *Chlamydomonas reinhardtii* cells have been studied as a standard microalgal cell model for transformation experiments, in this study, we evaluated the cellular uptake and cytotoxicity of linear and branched PEI in *C. reinhardtii* CC-1010, referred to as LPEI and BPEI, respectively (Figure 1(a and b)). However, both PEIs showed high toxicity due to oxidative stress. Here, we assumed that the suppression of oxidative stress associated with PEI decreased the cytotoxicity of PEI and increased its transformation efficiency. To suppress the oxidative stress associated with PEI, we developed PEI covalently conjugated with an antioxidant, 2,2,6,6-tetramethylpiperidine-1-oxyl (TEMPO); the conjugate was named as TEMPO-LPEI (Figure 1(c)). TEMPO can catalytically scavenge ROS, such as superoxide, hydroxyl radicals, and alkylperoxyl radicals [20]. In this study, we investigated the effect of the covalent modification of TEMPO into PEI on cytotoxicity and microalgal transformation with electroporation.

## 2. Materials and methods

### 2.1. Materials

BPEI and LPEI were purchased from Sigma-Aldrich (St Louis, Missouri, USA). The number average molecular weight of both BPEI and LPEI was 10 kDa. *N*-Hydroxysuccinimide (NHS), 1-ethyl-3-(3-dimethylaminopropyl) carbodiimide (EDC), 5-carboxyfluorescein succinimidyl ester, 2ʹ,7ʹ-dichlorodihydro-fluorescein diacetate (DCFH-DA), and 4-(2-iodoacetamido)-TEMPO were purchased from Sigma-Aldrich, DOJINDO (Kumamoto, Japan), Molecular Probes (Eugene, Oregon, USA), Cayman Chemical Company (Ann Arbor, Michigan, USA), and Tokyo Chemical Industry Co. Ltd. (Tokyo, Japan), respectively, and used without further purification.

### 2.2. C. reinhardtii *culture conditions*

*C. reinhardtii* strain CC-1010 was obtained from the Chlamydomonas Resource Center (St. Paul, Minnesota, USA). Cells were grown in a Tris acetate phosphate (TAP) medium at 25°C under continuous illumination provided by fluorescent lamps at a fluence rate of 50 μmol m^−2^ s^−1^ and bubbling with air containing 1.0% (v/v) CO_2_. The components of TAP have been described previously [21]. Cells were harvested during the exponential growth phase.

### 2.3. Preparation of fluorescein-labeled PEI and its cellular uptake

Fluorescein-labeled PEI (F-PEI) was prepared by reaction with 5-carboxyfluorescein in *N, N*-dimethylformamide. Briefly, 10 mg of PEI (1 μmol), 1.0 mg of 5-carboxyfluorescein (2.7 μmol), 1.0 mg of EDC (5.2 μmol), and 1.0 mg of NHS (8.7 μmol) were dissolved in 20 mL of *N, N*-dimethylformamide and stirred for 2 h at room temperature. The F-PEI solution was transferred into a pre-swollen membrane tube (molecular-weight cutoff size: 3,500, Spectra/Por, Rancho Dominguez, California, USA) and dialyzed for 24 h against 2 L of water.

After adding F-PEI to the algal cell cultures (1.0 × 10^6^ cells mL^−1^), the cells were cultured for 24 h in TAP medium at 25°C under continuous illumination provided by fluorescent lamps at a fluence rate of 50 μmol m^−2^ s^−1^ and bubbling with air containing 1.0% (v/v) CO_2_. The final concentration of F-PEI was 2 μM because the fluorescence signals of F-PEI could not be detected in the cells at the concentration below 2 μM. Microscope images for fluorescein (excitation: 400–490 nm, emission: 500–550 nm) and chlorophyll autofluorescence (excitation: 540–580 nm, emission 593–668 nm) were captured using a Zeiss Axio Observer Z1 microscope equipped with an AxioCam MRm digital camera (Zeiss, Gottingen, Germany). To analyze the intracellular uptake of F-PEIs semi-quantitatively, the mean fluorescence intensity (MFI) of fluorescein in the cells was measured using ImageJ software (National Institutes of Health, Bethesda, Maryland, USA).

### 2.4. Measurement of chlorophyll content in the cell culture

To determine the chlorophyll content in the cell culture, 200 µL aliquots of the culture were mixed with 800 µL of acetone. The mixture was kept on ice for 30 min to extract chlorophyll and then centrifuged at 4°C for 2 min at 15,000 × *g*. The absorbance values of the supernatants were measured at 645, 663, and 710 nm using a UV/Vis spectrophotometer (Ultrospec 3300 pro, GE Healthcare, Chicago, Illinois, USA). Total chlorophyll content was determined as described by Porra et al. [22].

### 2.5. Measurement of optical density

After adding PEI at final concentrations of 0, 25, 50, and 100 nM, the cells were cultured for 24 h in TAP medium at 25°C under continuous illumination provided by fluorescent lamps at a fluence rate of 50 μmol m^−2^ s^−1^ and bubbling with air containing 1.0% (v/v) CO_2._ The optical density in the culture was measured at a wavelength of 750 nm (OD_750_) using the UV/Vis spectrophotometer.

### 2.6. Analysis of oxidative stress in the cells

ROS levels in the cells were measured using ROS-sensitive probe, DCFH-DA, according to a previous report [23]. After cell uptake of non-fluorescent DCFH-DA, it is deacetylated by cellular esterases to a nonfluorescent compound, which is oxidized by ROS into fluorescent 2ʹ,7ʹ-dichlorofluorescein (DCF). Briefly, after adding PEI at final concentrations of 2 μM, the cells were cultured for 1 h in TAP medium at 25°C under continuous illumination provided by fluorescent lamps at a fluence rate of 50 μmol m^−2^ s^−1^ and bubbling with air containing 1.0% (v/v) CO_2._ Then, 1 μL of DCFH-DA solution (0.5 mg mL^−1^) was added to 100 μL of algal cells and mixed, followed by incubation for 30 min in the dark. Fluorescence signals of DCF in the cells was detected using a Gallios flow cytometer (Beckman Coulter, Brea, California, USA), quantifying 488 nm-excited fluorescence signals at 525/50 nm.

### 2.7. Synthesis of TEMPO-LPEI

TEMPO-LPEI was synthesized using LPEI and 4-(2-iodoacetamido)-TEMPO. Briefly, 10 mg of PEI and 24 mg of 4-(2-iodoacetamido)-TEMPO were dissolved in 2 mL of acetonitrile and stirred for 8 h at 60°C. The TEMPO-LPEI solution was transferred into a pre-swollen membrane tube (molecular-weight cutoff size: 3,500, Spectra/Por) and dialyzed for 48 h against 2 L of water, followed by freeze-drying. The electron spin resonance (ESR) spectra were recorded at room temperature on a JEOL-TE300 ESR spectrometer (JEOL, Tokyo, Japan) operating at 9.41 GHz. According to previous reports [24–27], the rotational correlation time (τ_c_) was calculated as

(1) $$\tau_{c}=6.6\times 10^{-10}\Delta H\left[{\left(h_{-1}/h_{+1}\right)}^{1/2}-1\right]$$

where *ΔH* is the peak-to-peak line width of the low-field line (in G), and h_−1_ and h_+1_ are the peak-to-peak heights of the low- and high-field lines, respectively.

### 2.8. Preparation of polyplexes with pDNA and TEMPO-LPEI

A pMF59 pDNA was amplified in *Escherichia coli*, and isolated and purified using a Qiagen Plasmid Plus Maxi Kit (Qiagen, Hilden, Germany). According to a previous report [28], TEMPO-LPEI/pDNA and LPEI/pDNA complexes were prepared by mixing the pDNA with TEMPO-LPEI and LPEI, respectively, at N/P ratios of 7.8, in TAP medium (N/P = molar ratio of PEI amino group to DNA phosphate).

### 2.9. Electroporation protocol

Cells were transformed with the pMF59 pDNA designed for the constitutive expression of the zeocin resistance gene [29]. The cells were collected by centrifugation at 1,000 × *g* for 5 min at room temperature and resuspended in TAP medium containing 40 mM sucrose to a final density of 1.0 × 10^8^ cells mL^−1^. Then, 2 µL of 200 µg mL^−1^ pDNA or its polyplex was added to 38 µL of the cell suspension, followed by incubation at room temperature for 2 h. The cell suspension was placed into an electroporation cuvette with a 2 mm gap (Nepa Gene, Chiba, Japan) and transformed by electroporation using a NEPA21 electroporator (Nepa Gene, Chiba, Japan). The parameters of the two poring pulses were set at 250 V with 8 ms length, 50 ms interval, and 40% decay rate, and those of the transfer pulses were set at five polarity-exchanged pulses of 20 V with 50 ms length, 50 ms interval, and 40% decay rate. After electroporation, the transformants were added to 10 mL of TAP medium containing 40 mM sucrose and incubated at 25°C for 24 h with gentle agitation under illumination at 2–3 μmol photons m^−2^ s^−1^. After incubation, the cells were collected by centrifugation at 1,000 × *g* for 5 min at 4°C and screened on TAP plates containing 1.5% agar and 5 µg mL^−1^ zeocin at 25°C under 80 μmol photons m^−2^s^−1^. After 7 days, colonies resistant to antibiotics appeared, and the number of colonies grown and transformed on the TAP agar plate with 5 mg L^−1^ zeocin was counted to calculate the transformation efficiency.

## 3. Results and discussion

### 3.1. Cytotoxicity of linear and branched PEI in C. reinhardtii

Since the green alga *C.* *reinhardtii* has been previously studied as a standard microalgal cell model for transformation experiments, *C. reinhardtii* CC-1010 was used in this study. However, the effects of PEI on intracellular uptake and toxicity in microalgal cells such as *C. reinhardtii* CC-1010 have not been known. Therefore, we first conducted an experiment to investigate whether PEIs were taken up by these algal cells. There are two types of commercially available PEIs, LPEI and BPEI. To observe their cellular uptake, both LPEI and BPEI were fluorescently labeled with carboxy-fluorescein, referred to as F-LPEI and F-BPEI, respectively. Since carboxy-fluorescein was reacted with the amines in the PEI *via* an amide bond, the number of amino groups in PEI would be changed, which may affect intracellular uptake when a large number of carboxy-fluorescein was reacted with PEI. To rule out the possibility, in this study, small amount of carboxy-fluorescein was reacted with PEI with approximately 250 amino groups. Although we did not measure the introduction amount of carboxy-fluorescein to PEI, based on the reaction condition, approximately one carboxy-fluorescein would be introduced to a PEI molecule with approximately 250 amino groups. Therefore, the effect of fluorescent labelling on the property of PEI can be ignored.

**Figure 2.**
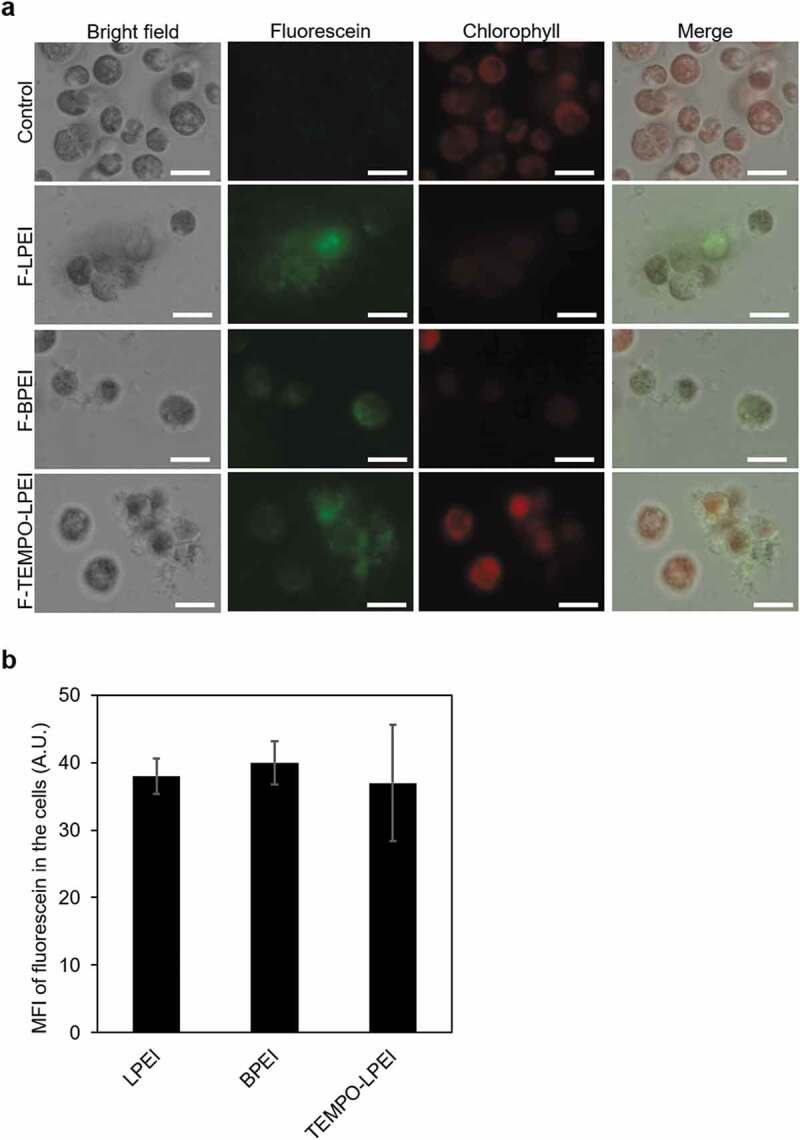
Microscopic observation of the cellular uptake of fluorescein-labeled PEIs and the autofluorescence of chlorophyll (a) Bright field and fluorescence images of *Chlamydomonas reinhardtii* cells that were cultured for 24 h after the addition of F-LPEI, F-BPEI, and F-TEMPO-LPEI at a concentration of 2 μM. In the fluorescence images, fluorescein and autofluorescent chlorophyll are shown as green and red, respectively. Scale bars = 20 μm. (b) The mean fluorescence intensity (MFI) of fluorescein in the cells. The intracellular uptake of F-PEIs was analyzed semi-quantitatively using the fluorescent microscope images that are focused on the inside of the cells

**Figure 3.**
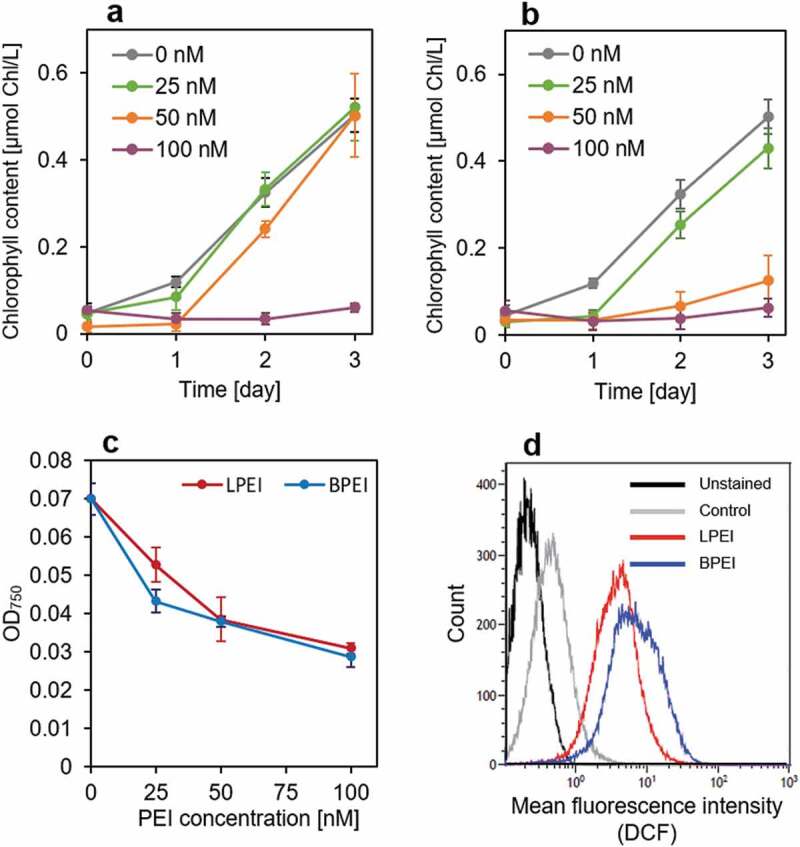
Cytotoxicity of LPEI and BPEI. (a,b) Chlorophyll content in the culture on day 1, 2, and 3 after the addition of (a) LPEI and (b) BPEI at concentrations of 0, 25, 50, and 100 nM. Chlorophyll is one of the cellular components in the microalgae that can be used for estimating cell proliferation. Each value represents the mean ± standard deviation (SD) of four independent experiments. (c) Optical density of the culture at 750 nm (OD_750_) 24 h after the addition of LPEI or BPEI at concentrations of 0, 25, 50, and 100 nM. Each value represents the mean ± SD of four independent experiments. (d) Flow cytometry histogram of the cells stained by ROS-sensitive probe, DCFH-DA. Black: unstained cells; gray: stained cells as a control; red: stained cells 1 h after adding 2 μM LPEI; blue: stained cells 1 h after adding 2 μM BPEI

After F-LPEI or F-BPEI was added to the culture at a concentration of 2 µM, fluorescence signals of F-LPEI and F-BPEI were detected in the cells (Figure 2(a)). The MFI of fluorescein in the cells was measured using the ImageJ software; the MFIs in the LPEI- and BPEI-treated cells were 39 ± 2.6 and 40 ± 3.2 (arbitrary unit, A.U.), respectively (Figure 2(b)). This indicated that F-LPEI and F-BPEI were taken up by the cells to the same extent. In addition, after the addition of F-LPEI or F-BPEI, the autofluorescence of chlorophyll appeared to be decreased compared to the control, suggesting that the cells were damaged (Figure 2(a)). Here, we investigated the cell proliferation after adding the PEIs to the cell culture. Measurements of chlorophyll content and OD_750_ are commonly used methods for assessing microalgal cell proliferation. Chlorophyll is one of the cellular components in the microalgae [22]. However, the intracellular chlorophyll content is changed by cell state; for example, the chlorophyll content is dramatically decreased when the cells have damages [30]. On the other hand, OD_750_ is widely used to monitor microalgal growth, which is inexpensive and reliable method. Measurement at 750 nm avoids the absorption of light by cellular pigments such as chlorophyll and carotenoids. However, OD_750_ measurement also has the major drawback because it is affected by cell size, cell aggregate, and so on [31]. Thus, both methods have the advantages and disadvantages. To evaluate accurate cell proliferation, in this study, both methods were used. First, the time course of chlorophyll content in the culture after adding the lower concentrations of PEIs was examined (Figure 3(a and b)). When the cells were cultured for 3 days in the absence of PEI, the chlorophyll content in the culture was gradually increased, thus indicating cell growth. In contrast, after the addition of 100 nM LPEI or BPEI, the chlorophyll content did not increase. This result demonstrates the toxicity of both PEIs to the cells. At the PEI concentration of 50 nM, the chlorophyll content was not increased on day 1 after the LPEI or BPEI addition, while the increased chlorophyll content in the LPEI-treated cell culture was higher than BPEI after day 2 (Figure 3(a and b)). This indicates that LPEI is less cytotoxic than BPEI. Probably, on day 1 after the addition of 50 nM LPEI, the cells were stressed by LPEI, yet after day 2, they may have adapted to the stress and proliferated. On the other hand, the BPEI-treated cells could not grow even after day 2 due to the severe damages. We also measured OD_750_ of the culture. The OD_750_ was measured on day 1 after the PEI addition, and dramatically decreased with increasing concentrations of both LPEI and BPEI (Figure 3(c)). These results indicates that cell proliferation was suppressed by the addition of both PEIs to the culture.

To determine the reason behind PEI toxicity, we focused on oxidative stress. In our previous study, we found that the cellular uptake of PEI induces an excessive production of ROS in *Haematococcus pluvialis* NIES-144 cells, leading to the accumulation of astaxanthin [23]. In this study, we investigated the effects of both LPEI and BPEI on oxidative stress in the cells using ROS-sensitive probe, DCFH-DA, in order to understand the mechanism of cytotoxicity. The DCFH-DA was added at 1 h after the addition of PEI in order to evaluate the intracellular oxidative stress under the condition without cell growth. In the present condition, at the PEI concentration of 2 μM, the fluorescence intensity of DCF significantly increased in cells treated with both LPEI and BPEI (Figure 3(d)). Comparing LPEI and BPEI, it was found that the level of BPEI-induced ROS production was higher than that of LPEI, which was consistent with the higher toxicity of BPEI (Figure 3(a and b)). Therefore, the less toxic LPEI was used for the transformation experiments.

### 3.2. Cytotoxicity of TEMPO-LPEI and its application in microalgal transformation

**Figure 4.**
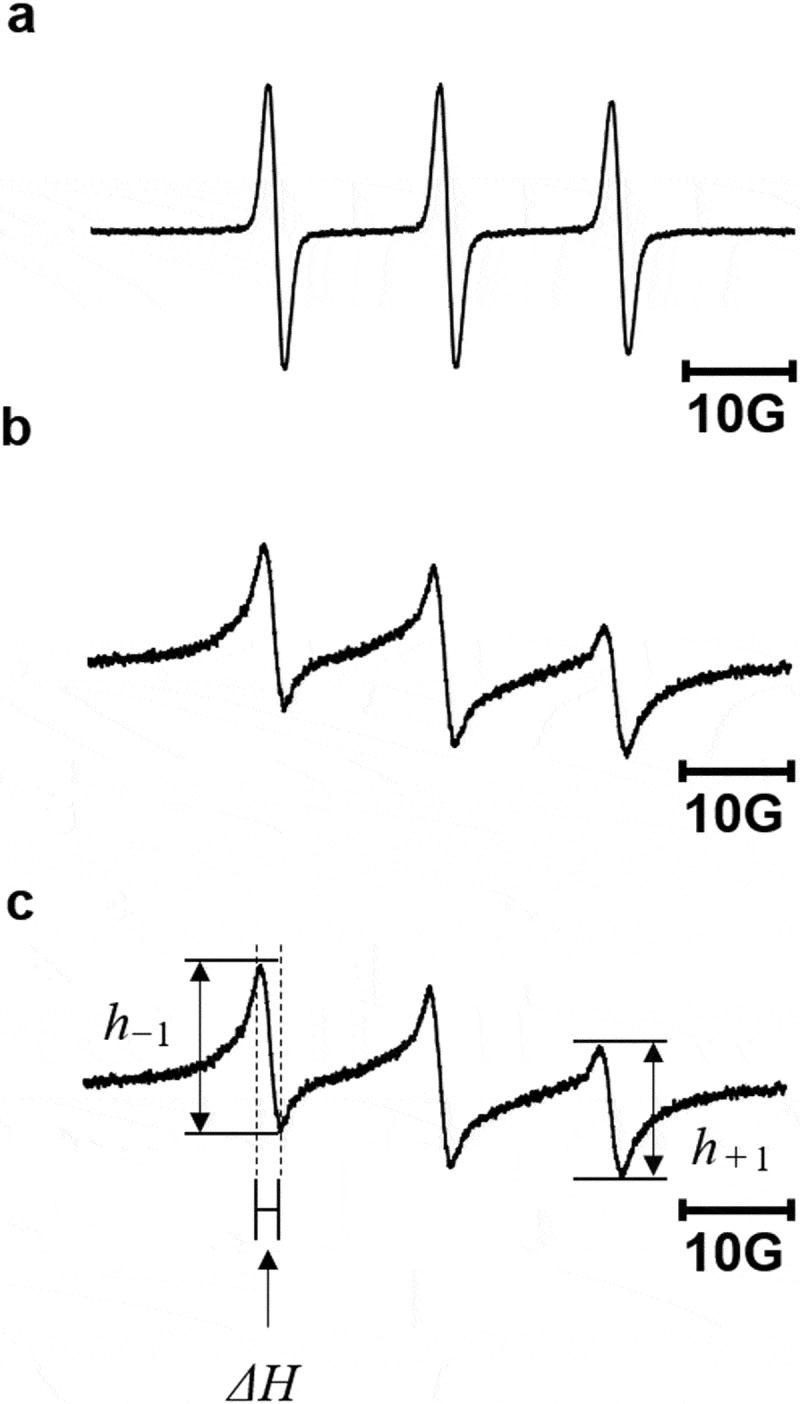
ESR spectra of 4-(2-iodoacetamido)-TEMPO and TEMPO-LPEI. (a) ESR spectrum of 4-(2-iodoacetamido)-TEMPO. (b) ESR spectrum of TEMPO-LPEI. (c) The peak-to-peak height (h_−1_ and h_+1_) and width of line (*ΔH*) are shown in the ESR spectrum of TEMPO-LPEI

To suppress the oxidative stress associated with LPEI, we developed LPEI covalently conjugated with the antioxidant, TEMPO, referred to as TEMPO-LPEI (Figure 1(c)). TEMPO possesses an unpaired electron, which can be used as an ESR probe [20]. Low-molecular weight 4-(2-iodoacetamido)-TEMPO showed sharp triplet ESR signals corresponding to an interaction between the ^14^N nuclei and the unpaired electron (Figure 4(a)). Although ESR spectrum of TEMPO-LPEI also showed triplet signals (Figure 4(b)), its rotational correlation time (τ_c_), which indicates the rotational mobility of the ESR probe, were different from that of 4-(2-iodoacetamido)-TEMPO (Figure 4(c) and Equation 1). The τ_c_ values of TEMPO-LPEI and 4-(2-iodoacetamido)-TEMPO were 2.88 × 10^−10^ and 0.91 × 10^−10^ s, respectively. A higher τ_c_ value indicates a lower mobility of the TEMPO moieties, which indicates the cross-linkage of TEMPO molecules on LPEI. In addition, the amount of TEMPO in TEMPO-LPEI was measured from the area of the ESR spectrum; approximately two TEMPO molecules were covalently linked in approximately 250 amino groups of LPEI. The reaction of PEI with 4-(2-iodoacetamido)-TEMPO changes the classification of amines in PEI. For example, when most of the secondary amines mainly present in PEI are converted to tertiary, or quaternary amines by the reaction with 4-(2-iodoacetamido)-TEMPO, the property of the PEI such as the cellular uptake and the formation of polyplexes with pDNA would be changed. In this study, since small amount of TEMPO molecules was covalently with LPEI with approximately 250 amino groups, the changing property of PEI by the TEMPO introduction can be ignored.

The cellular uptake of TEMPO-LPEI was investigated to evaluate the effect of the covalent modification of TEMPO. After F-TEMPO-LPEI was added to the culture at a concentration of 2 µM, fluorescence signals of F-TEMPO-LPEI were detected in the cells (Figure 2(a)). The MFI of fluorescein in the F-TEMPO-LPEI-treated cells was 37 ± 8.6 (A.U.), as measured by the ImageJ software, which was not significantly different from that in the cells treated with F-LPEI (Figure 2(b)). This indicated that the covalent modification of approximately two TEMPO molecules on LPEI did not affect the cellular uptake of PEI. In addition, clear autofluorescence of chlorophyll was detected after the addition of F-TEMPO-LPEI (Figure 2(a)).

**Figure 5.**
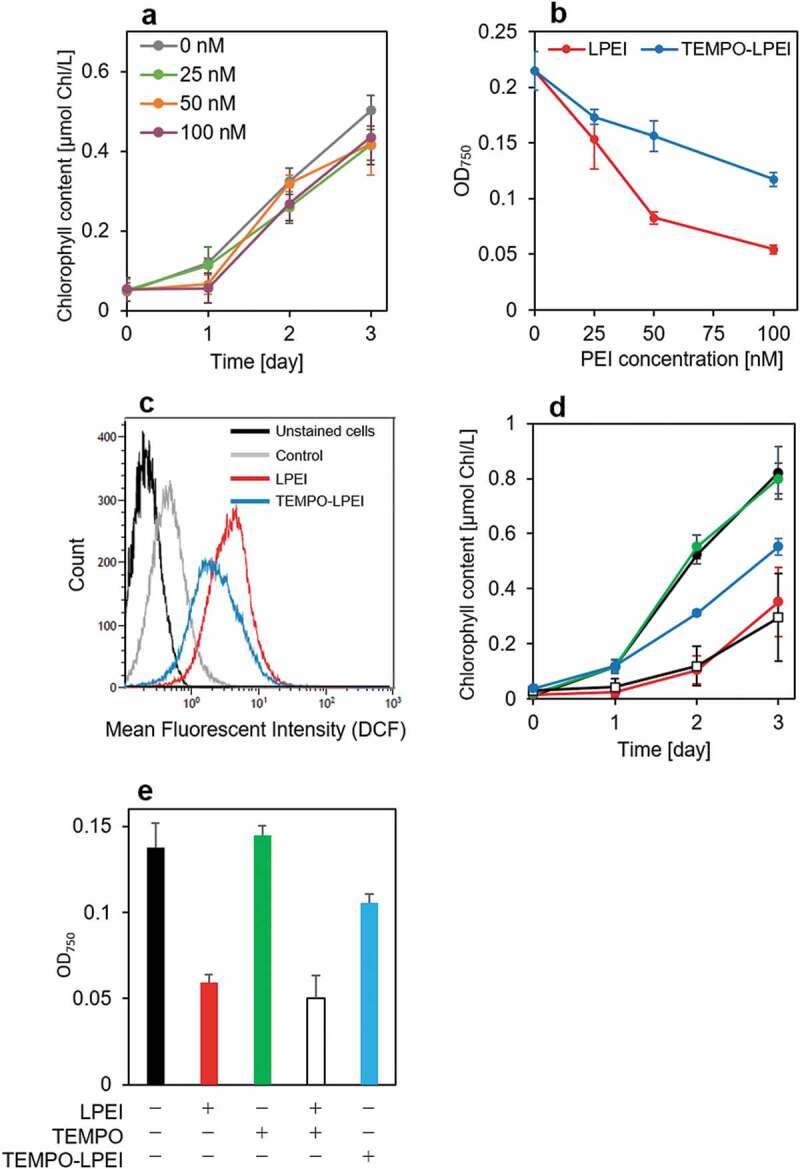
Effect of TEMPO-LPEI on cytotoxicity. (a) Chlorophyll content in the culture on day 1, 2, and 3 after the addition of TEMPO-LPEI at concentrations of 0, 25, 50, and 100 nM. Chlorophyll is one of the cellular components in the microalgae that can be used for estimating cell proliferation. Each value represents the mean ± SD of four independent experiments. (b) Optical density of the culture at 750 nm (OD_750_) 24 h after the addition of LPEI or TEMPO-LPEI at concentrations of 0, 25, 50, and 100 nM. Each value represents the mean ± SD of four independent experiments. (c) Flow cytometry histogram of the cells stained by ROS-sensitive probe, DCFH-DA. Black: unstained cells; gray: stained cells as a control; red: stained cells 1 h after adding 2 μM LPEI; blue: stained cells 1 h after adding 2 μM TEMPO-LPEI. (d) Chlorophyll content in the culture on day 1, 2, and 3 after the addition of TAP medium (black), LPEI (red), 4-(2-iodoacetamido)-TEMPO (green), mixture of LPEI and 4-(2-iodoacetamido)-TEMPO (white), and TEMPO-LPEI (blue). The final concentrations of 4-(2-iodoacetamido)-TEMPO, LPEI, and TEMPO-LPEI in the culture were 200, 100, and 100 nM, respectively. Each value represents the mean ± SD of four independent experiments. (e) Optical density of the culture at 750 nm (OD_750_) 24 h after the addition of TAP medium (black), LPEI (red), 4-(2-iodoacetamido)-TEMPO (green), mixture of LPEI and 4-(2-iodoacetamido)-TEMPO (white), and TEMPO-LPEI (blue). Final concentrations of 4-(2-iodoacetamido)-TEMPO, LPEI, and TEMPO-LPEI in the culture were 200, 100, and 100 nM, respectively. Each value represents the mean ± SD of four independent experiments

To check the cytotoxicity of TEMPO-LPEI, the chlorophyll content and OD_750_ of the cultures were measured. As shown in Figure 5(a), the chlorophyll content in the cell culture was increased even after the addition of TEMPO-LPEI at a concentration of 100 nM. In addition, the suppression of cytotoxicity of PEI by the installation of TEMPO was also shown by the results of OD_750_ (Figure 5(b)). Furthermore, flow cytometry results showed that the covalent modification of TEMPO on LPEI reduced the production of intracellular ROS (Figure 5(c)). Thus, by introducing TEMPO molecules into PEI, the toxicity of PEI was dramatically reduced. We investigated the effect of the covalent conjugation of TEMPO molecules on LPEI on cytotoxicity by measuring both chlorophyll content and OD_750_ (Figure 5(d and e)). Unlike TEMPO-LPEI, the mixture of PEI and 4-(2-iodoacetamido)-TEMPO was unable to reduce the toxicity of PEI. Low-molecular-weight TEMPO cannot efficiently scavenge the ROS generated in the vicinity of PEI because it diffuses freely in the medium and in the cells. In contrast, due to the covalent conjugation of TEMPO molecules on LPEI, TEMPO-LPEI can efficiently scavenge the ROS generated in the vicinity of PEI after the cellular uptake. This result indicates the importance of the covalent conjugation of antioxidants on polycations.

Further, we investigated the effect of polyplexes of TEMPO-LPEI and pDNA on the transformation of microalgal cells using electroporation. TEMPO-LPEI/pDNA and LPEI/pDNA polyplexes were prepared by mixing pDNA with TEMPO-LPEI and LPEI, respectively, at N/P ratios of 7.8 in TAP medium at pH 7.0. Since approximately 20% of the amino groups in PEI were protonated and were positively charged in TAP medium at pH 7.0, according to previous reports [32], the molar ratio of negatively charged DNA phosphate to positively charged amino groups in the polyplexes was 0.61 in TAP medium, indicating that the polyplexes were slightly positively charged.

**Figure 6.**
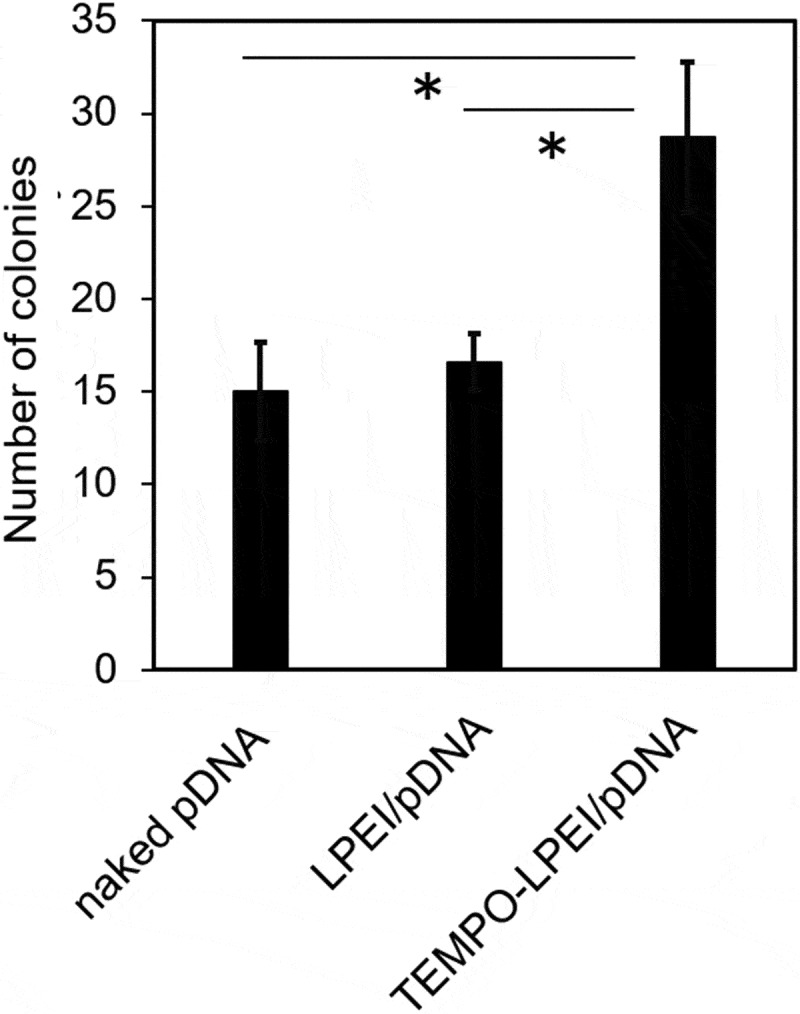
Colony number of *Chlamydomonas reinhardtii* after transformation through electroporation with naked pDNA, LPEI/pDNA, and TEMPO-LPEI/pDNA. Each value represents the mean ± SD of three independent experiments. *P < 0.05

To investigate the effect of polyplexes on transformation, electroporation was carried out at 2 h after adding the polyplex or naked pDNA into the cell culture. Positive transformant colonies appeared after 7 days on TAP agar plates containing 5 mg L^−1^ zeocin. The number of colonies grown and transformed on the TAP agar plate with 5 mg L^−1^ zeocin was counted to calculate the transformation efficiency (Figure 6). No colonies appeared by the addition of no pMF59 pDNA, LPEI, or TEMPO-LPEI in the present condition (data not shown). When naked pMF59 pDNA was used as a control, the average number of colonies was 15. When polyplexes of LPEI/pDNA were used, the average number of colonies was 17. In contrast, when the polyplexes with TEMPO-LPEI were used, the average number of colonies doubled to 29, and the transformation efficiency markedly increased. Although there is no significant difference in Figure 6, the number of colonies was slightly higher with LPEI/pDNA than that with naked pDNA. The formation of pDNA polyplex with polycation causes not only the neutralization of negative charge of pDNA but also the compaction of pDNA into polyplexes. By this feature of polyplex, probably, the efficiency of pDNA delivery in the microalgal cells was enhanced, increasing the transformation efficacy. However, the cellular uptake of PEI itself causes oxidative stress that decreases the transformation efficiency. In contrast, TEMPO-PEI would enhance the efficacy of pDNA delivery without the increased oxidative stress by efficiently scavenging ROS generated by PEI with covalently conjugated TEMPOs, and improve the efficiency of transformation. Further improvement of transformation efficiency can be expected by examining the amount of TEMPO introduced and N/P ratio of the polyplexes with TEMPO-LPEI/pDNA.

In summary, this study demonstrates that PEI-induced intracellular oxidative stress and toxicity can be reduced through the covalent conjugation of TEMPO on LPEI. In addition, by forming the polyplexes of TEMPO-LPEI and pDNA, transformation efficiency can be further improved. This study provides guidelines for designing materials that reduce oxidative stress caused by polycations and clearly shows the usefulness of the polyplexes with non-toxic polycations for efficient transformation for microalgal cells. To the best of our knowledge, this is the first study using the polyplex with polycations/pDNA for the microalgal transformation, and has the potential to be a breakthrough method for the transformation of microalgal cells.
